# Converging Mechanisms of Vascular and Cartilaginous Calcification

**DOI:** 10.3390/biology13080565

**Published:** 2024-07-26

**Authors:** Simona R. Gheorghe, Alexandra M. Crăciun, Tamás Ilyés, Ioana Badiu Tisa, Lucia Sur, Iulia Lupan, Gabriel Samasca, Ciprian N. Silaghi

**Affiliations:** 1 Department of Medical Biochemistry, Iuliu Hatieganu University of Medicine and Pharmacy, 400349 Cluj-Napoca, Romania; gheorghe.simona@umfcluj.ro (S.R.G.); acraciun@umfcluj.ro (A.M.C.); tamas.ilyes@umfcluj.ro (T.I.); silaghi.ciprian@umfcluj.ro (C.N.S.); 2 Department of Pediatrics III, Iuliu Hatieganu University of Medicine and Pharmacy, 400217 Cluj-Napoca, Romania; ioana.badiu@umfcluj.ro; 3 Department of Pediatrics I, Iuliu Hatieganu University of Medicine and Pharmacy, 400370 Cluj-Napoca, Romania; sur.maria@umfcluj.ro; 4 Department of Molecular Biology, Babes-Bolyai University, 400084 Cluj-Napoca, Romania; iulia.lupan@ubbcluj.ro; 5 Department of Immunology, Iuliu Hatieganu University of Medicine and Pharmacy, 400162 Cluj-Napoca, Romania

**Keywords:** vascular calcification, cartilaginous calcification, calcification mechanisms

## Abstract

**Simple Summary:**

The normal calcification process is essential for bone formation, but calcification found elsewhere can result in cardiovascular and joint complications. Ectopic calcification, influenced by factors such as hormonal imbalance and oxidative stress, can impact soft tissues and becomes more prevalent with age and chronic illness. The stiffening of soft tissues due to calcification is a complex process influenced by cell type, interactions, the extracellular matrix, and mechanical forces. Understanding these mechanisms is vital for developing treatments for calcification-related complications. This review explores the similarities between vascular and cartilage calcifications, offering insights into both normal and pathological aspects.

**Abstract:**

Physiological calcification occurs in bones and epiphyseal cartilage as they grow, whereas ectopic calcification occurs in blood vessels, cartilage, and soft tissues. Although it was formerly thought to be a passive and degenerative process associated with aging, ectopic calcification has been identified as an active cell-mediated process resembling osteogenesis, and an increasing number of studies have provided evidence for this paradigm shift. A significant association between vascular calcification and cardiovascular risk has been demonstrated by various studies, which have shown that arterial calcification has predictive value for future coronary events. With respect to cartilaginous calcification, calcium phosphate or hydroxyapatite crystals can form asymptomatic deposits in joints or periarticular tissues, contributing to the pathophysiology of osteoarthritis, rheumatoid arthritis, ankylosing spondylitis, tendinitis, and bursitis. The risk factors and sequence of events that initiate ectopic calcification, as well as the mechanisms that prevent the development of this pathology, are still topics of debate. Consequently, in this review, we focus on the nexus of the mechanisms underlying vascular and cartilaginous calcifications, trying to circumscribe the similarities and disparities between them to provide more clarity in this regard.

## 1. Introduction

The calcification or mineralization process is actively managed by cells involved in the formation, arrangement, and maintenance of the extracellular matrix (EM). This process leads to the deposition and buildup of inorganic substances such as calcium, phosphate, magnesium, and bicarbonate. Physiologically, mineralization is limited to bones, teeth, growth plates, and deep layers of articular cartilage and is controlled by both inhibitory and stimulatory factors [1,2]. In pathological conditions, mineralization can also occur in soft connective tissues. Ectopic or extraskeletal calcification may stem from genetic mutations (e.g., ABCC6, ATP-binding cassette C subfamily member 6 in pseudoxanthoma elasticum; MGP, matrix Gla protein in Keutel syndrome; FAM20A, family with sequence similarity 20 member A in amelogenesis imperfect) [3,4] or from acquired chronic illnesses (e.g., chronic kidney disease, diabetes, atherosclerosis). The cardiovascular system, specifically the vessels and valves, and the joints are primarily impacted by pathological mineralization, leading to disease and clinical complications. This is one of the major reasons for illness and death in the civilized world among the aging population, as a pro-osteogenic environment gradually develops [5,6,7].

The occurrence of ectopic calcification can be attributed to various mechanisms, including disruptions in hormonal balance [8], uncontrolled angiogenesis and vascular repair processes [9], and irregular extracellular nucleotide metabolism [10]. Recently, special attention has been given to the discharge of matrix vesicles (MVs) for the development and advancement of mineralization in soft connective tissues. Hard tissues, including bones and teeth, are a fundamental part of the body, and their formation and homeostasis are critically regulated by MV-mediated mineralization. MVs have been studied for 50 years since they were first observed using electron microscopy. Although research progress has been hampered by various technical barriers, there have been great advancements in our understanding of the intracellular biosynthesis of MVs. Mitochondria and lysosomes are now considered key players in MV formation. The involvement of damaged mitochondria removal, vesicles formed from mitochondrial membranes, and mitochondria–lysosome interactions have been suggested as potential detailed mechanisms of the intracellular pathway of MVs. Their main secretion pathway may involve active transport and discharge in the extracellular space, in addition to the traditionally understood mechanism of budding from the outer plasma membrane. This basic knowledge of MVs should be strengthened by novel microscopic technologies, together with basic cell processes, such as autophagy and interorganelle interactions. In the field of tissue regeneration, extracellular vesicles such as exosomes are gaining interest as promising tools for cell-free bone and periodontal regenerative therapy. MVs, which are recognized as a special type of extracellular vesicle, could be another potential alternative [11,12].

The objective of this review is to provide an overview of the extraskeletal calcification mechanisms underlying the similarities and disparities between vascular and cartilaginous calcifications and to indicate a few possible therapeutic approaches.

## 2. Types and Characteristics of Ectopic Calcifications: Correspondence with Osteogenesis

Calcification is one of the final stages of osteogenesis (bone formation or ossification), but a more comprehensive term is mineralization, because crystal precipitation occurs in the form of calcium phosphate. Osteogenesis is a physiological process that occurs in bone and cartilage and can occur through two processes: membrane ossification (intramembranous or desmal) and cartilage ossification (endochondral or enchondral). These two processes are carried out until the end of the growth period, whereas throughout life, the bone undergoes a remodeling process in which the balance between bone resorption and bone formation is maintained by local factors (e.g., cytokines and the concentration of extracellular calcium and phosphorus) and systemic factors (e.g., hormones and mechanical stress). On the other hand, there are ectopic calcifications (heterotopic or dystrophic, often found in blood vessels and soft tissues) representing pathological calcifications because of extraskeletal bone tissue metaplasia. It is interesting to trace how physiological ossification corresponds with pathological calcifications, as well as the paradox of osteoporosis, in which ectopic calcification overlaps with bone demineralization [13].

Both types of ossification occur in vascular calcification (VC), depending on the pathology: intramembranous, which does not have cartilage as the starting point, but also endochondral ossification, which uses cartilage as a template. The classification of VC is based on histological and etiological criteria. Histologically, they are categorized as osteomorphic, chondromorphic, or amorphic. Etiologically, they can also be classified as metastatic and dystrophic arterial calcification [14]. Cardiovascular calcification can develop in distinct areas, such as the intima or media of vessel walls or heart valves, or can be generalized (calciphylaxis). Brief characteristics are summarized below.

- Intimal calcification is an endochondral ossification process in which type II collagen is mineralized by calcium deposits.
- Intramembranous ossification is responsible for media calcifications
- Dystrophic calcification appears in necrotic tissues or as a reaction to tissue destruction leading to valvular calcification.
- Vascular calciphylaxis or calcific uremic arteriolopathy is a systemic process characterized by diffuse calcification in the media of small and medium arteries or arterioles and intimal proliferation resulting in tissue necrosis.

Intimal calcification arises in the context of atherosclerosis. During the process of plaque formation, vascular smooth muscle cells (VSMCs) migrate from the media to the intima in response to various stimuli (i.e., chemotactic and mitogenic factors released by the vascular endothelium, activated macrophages, and neighboring VSMCs) [15]. Furthermore, VSMCs proliferate, then begin to accumulate lipids and finally initiate EM synthesis. Calcification occurs in the core of atherosclerotic plaques in close proximity to inflammatory cell infiltration and apoptotic remnant cells. The progression of this mechanism is controlled by certain inflammatory substances, such as lipoproteins and cytokines, which are typically found in the atheromatous parts of plaques [16,17].

Medial calcifications appear independently of intimal calcification and atherosclerosis. Calcification of the media of peripheral arteries is known as Mönckeberg’s sclerosis and usually occurs in patients with diabetes mellitus (DM) and in elderly individuals. Although initially thought to be harmless and not connected to narrowing or blood clot formation within the blood vessel, medial artery calcification is now known to be a redoubtable condition associated with increased cardiovascular death rates and an increased risk of limb amputation in patients with both type 2 DM and end-stage kidney disease. The causes of medial calcification are diverse and differ depending on the specific reasons. For example, arterial calcification in diabetic patients is mainly composed of hydroxyapatite crystals (HC), while whitlockite appears to be the primary component in cases of vitamin D toxicity [18]. Both elastin and VSMCs are involved in the process of media calcification [19]. Therefore, VSMCs are associated with both intimal and medial calcification.

Persy et al. [20] and Towler [21] described the similarities and structural differences between osteogenesis (as a model of physiological mineralization) and vascular calcification (as a model of pathological calcification), which are summarized in Table 1.

## 3. Mechanisms of Vascular and Cartilaginous Calcifications

**Figure 1:**
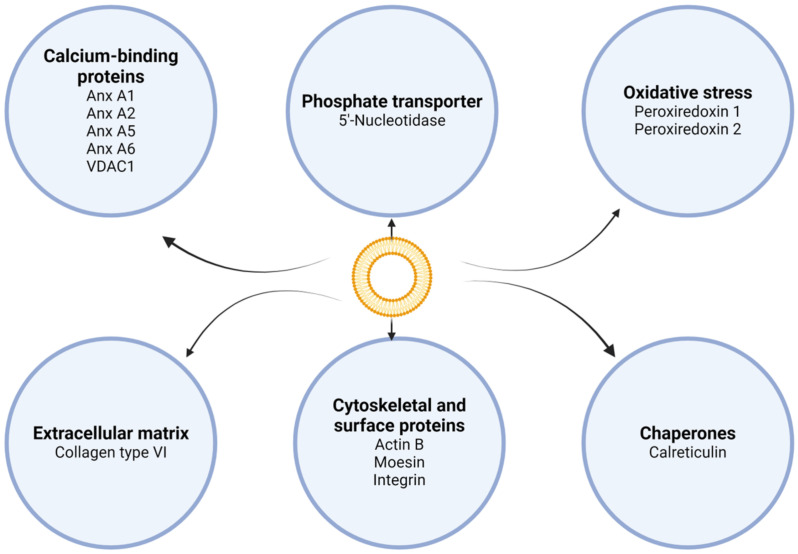
Proteins identified in MVs. Each circle represents a protein type or family (bold) and the protein name or family member. Abbreviations: Anx, annexin; VDAC1, voltage-dependent anion channel 1.

The previous understanding that VC is a passive process has been updated, and it is now recognized as an active, cell-mediated, and regulated complex phenomenon that occurs due to an imbalance between processes that encourage calcium deposition in the vascular wall and those that restrict it [22]. The initiation of mineralization in arteries and cartilage is governed by MVs derived from chondrocytes and VSMCs [23,24,25,26]. Figure 1 shows a few proteins identified in MVs.

MVs are circumscribed membrane nanoparticles (30–2000 nm) released by cells involved in the deposition of HC, budding cells, or disintegrated cells, and are able to increase the concentration of Ca^2+^ and PO_4_ above the solubility limit, thereby promoting the precipitation of calcium phosphate [23]. Several researchers have suggested that MVs derived from VSMCs are similar to exosomes (30–150 nm) derived from endosomes [27], while other studies on chondrocytes and osteoblasts indicate that MVs originate from the cellular plasma membrane budding [28,29] and apoptotic bodies (ABs) (500–2000 nm) discharged by dying cells [30,31,32,33]. The biogenesis of MVs is still being investigated, as it may vary depending on the type of cell and the chemical and osmotic characteristics of the EM. MVs with similar structures and compositions to those of skeletal MVs have been found in soft connective tissues of the cardiovascular system [34,35], suggesting that the mechanisms of extraskeletal calcification resemble those involved in normal skeletal development [36]. MVs are produced in articular cartilage or epiphyseal growth plates by hypertrophied chondrocytes [24], as well as in calcified arteries and atherosclerotic plaques, where VSMCs release MV-like structures with functions similar to those of MVs produced by chondrocytes [26].

The beginning of mineralization seems to be performed by MVs in cooperation with collagen fibrils: once the first crystals of calcium phosphate merge to form the mineralized front, the following steps, represented by the multiplication and growth of the crystals, are carried out independently of MVs [19].

The MV membrane released by chondrocytes located in the epiphyseal plate contains alkaline phosphatase (ALP), which hydrolyzes phosphoric acid esters from different substrates, thereby leading to an increase in intravesicular inorganic phosphate (Pi) (the intravesicular transport of Pi is mediated by Na-dependent cotransporters, especially Na-independent transporters) [37,38]. The process is similar in osteoblasts. The takeover of calcium via MVs released by chondrocytes is mediated by annexin (ANX), a family of proteins that bind phospholipids in the presence of Ca^2+^. ANX type V, which is expressed by the chondrocytes of the epiphyseal growth plate, binds to type II and X collagen molecules, resulting in the influx of calcium into vesicles. Only hypertrophic chondrocytes contain calcium levels high enough to allow ANX anchoring to the cell membrane [24,39]. Moreover, in vivo, ANX type VI was found to be abundant at sites of vascular mineralization, and VSMC mineralization was decreased by siRNA-mediated depletion of ANX type VI. Additionally, studies using biotin cross-linking and flow cytometry have indicated that ANX type VI moves to the plasma membrane when calcium levels are elevated in vitro and creates ANX type VI–PS nucleation complexes within MVs [33].

**Figure 2:**
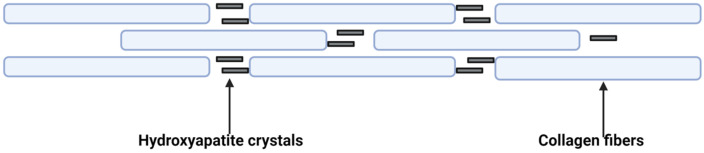
The deposition pattern of hydroxyapatite crystals. Hydroxyapatite crystals accumulate in the spaces between collagen fibers.

MVs that are competent for mineralization include a nucleation core composed of amorphous calcium phosphate, phosphatidylserine, and ANX [40], but there are also MVs incompetent for mineralization due to the lack of a nucleation core. Competent MVs are capable of increasing the local levels of Ca^2+^ and Pi above the physiological solubilization threshold, which leads to the initiation of crystal nucleation. The precise location of crystal formation (intra/extravesicular) and the nature of the first deposited crystal are still debated. Conversely, after the first nucleation, the following processes are well defined: precipitated crystals (commonly referred to as HCs) that bind to collagen fibrils are nonstoichiometric and have a carbonate–apatite Ca^2+^-deficient composition and organization that varies with the age of the crystal. HCs are rods distributed along collagen fibers (see Figure 2). Collagen arrangement is realized in such a manner as to give rise to spaces within an ordered network. In these spaces, HCs are arranged with their long axis parallel to the collagen fiber. The nucleation of collagen fibers begins at their interface. Finally, crystal aging causes organization, loss of acid phosphates, and incorporation of carbonate ions [41].

**Figure 3:**
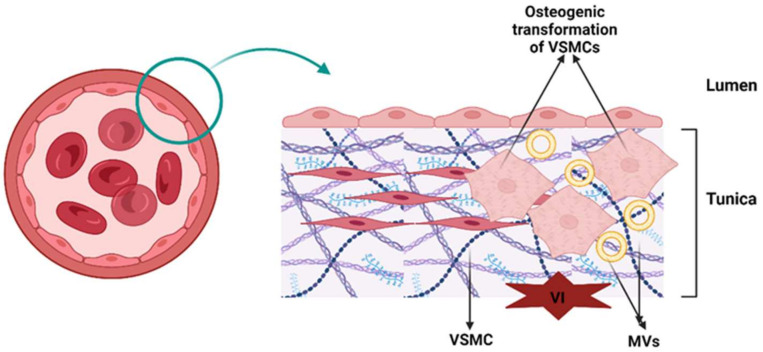
Vascular calcification. VSMCs stimulate MV discharge for mineralization. After a mechanical issue (vascular injury), VSMCs undergo osteogenic transformation and stimulate the release of MVs to complete the mineralization process. Abbreviations: VSMCs, vascular smooth muscle cells; MVs, matrix vesicles; VI, vascular injury.

There are several mechanisms that underlie ectopic calcification, acting either independently or in conjunction [42,43]: (A) induction of osteoblastic–chondrogenic differentiation of VSMCs; (B) apoptosis; (C) the presence of circulating nucleation complexes; and (D) imbalance/decline in inhibitors.

(A) Induction of osteoblastic–chondrogenic differentiation of VSMCs. In VSMCs of calcified arteries and atherosclerotic plaques, the mineralization process is as follows. VSMCs release MV-like structures, and under conditions of high concentrations of Ca^2+^ and Pi in the surrounding environment, VSMCs are stimulated to discharge competent MVs for mineralization [25,26]. VSMCs express ALP throughout chondrogenic differentiation, thereby increasing the local Pi level. ALP was also found in MVs released by VSMCs [26,42]. Moreover, in calcified arterial walls, as well as in VSMC culture, HCs had approximately the same pattern of composition during crystal growth as in the case of chondrocytes or bone [44,45]. Figure 3 summarizes the processes of vascular calcification.

Several factors that can trigger osteoblastic or chondrogenic metaplasia of VSMCs and calcification of vascular EM have been described as follows.

1. Factors that stimulate chondrogenic transdifferentiation (metaplasia) of VSMCs include mechanical factors (e.g., strain and elongation) [46,47], hypoxia [48,49], Pi (which stimulates the expression of the core binding factor alpha1, a transcription factor associated with osteoblast differentiation and EM mineralization of VSMCs) [50], cytokines, growth factors (e.g., transforming growth factor β (TGF-β), which stimulates the mineralization of VSMCs, and tumor necrosis factor α (TNF-α), which promotes the final differentiation of VSMCs into chondrocyte-like cells) [51,52] and bone morphogenic protein (BMP) 2, which stimulates osteogenic and chondrogenic differentiation [53].
2. Factors that promote osteogenic metaplasia include BMP-2 and BMP-4 (while BMP-7 prevents this differentiation) [54], core binding factor alpha 1 [43], Ca^2+^ and Pi [19], and ALP (which are highly expressed by VSMCs of calcified media vessels) [55]. ALP uses inorganic pyrophosphate (PPi), which is an inhibitor of EM mineralization, as a substrate. Other factors that promote osteogenic transformation are reactive oxygen species (ROS) (i.e., oxidized low-density lipoprotein cholesterol (LDL-C) stimulates the expression of BMP-2 and core binding factor alpha 1] [56]; vitamin D (1,25 dihydroxy vitamin D stimulates ALP activity and exacerbates dystrophic calcifications) [57]; warfarin (a vitamin K antagonist promotes vascular calcification by inhibiting γ-carboxylation of matrix Gla protein (MGP)) [58]; glucocorticoids (e.g., dexamethasone) mediate osteoblast differentiation, thus promoting calcification by suppressing calcification inhibitory molecules) [59]; leptin (a hormone involved in appetite regulation that mediates ectopic calcification by binding and activating β-adrenergic receptors on osteoblasts and increasing the levels of oxidative stress in aortic endothelial cells) [60,61]; and apoptosis (apoptotic remnants of foamy cells, debris VSMCs and MVs increase the local concentration of calcium phosphate, thus promoting an environment suitable for mineral nucleation) [35,43].

(B) Apoptosis. The association between apoptosis and vascular or soft tissue calcification has been widely documented. Vascular calcification is less common because the cellular debris of VSMCs is removed by phagocytic cells, and its clearance may be inhibited under certain conditions, leading to the accumulation of ABs [62,63]. Depending on local conditions, ABs undergo mineralization [64]. Kockx et al. [65] demonstrated that atherosclerotic plaques contain residues of VSMCs and Abs. During apoptosis, Ca^2+^ and Pi stored in mitochondria and sarcoplasm are incorporated into ABs and contribute to the formation of calcium phosphate crystals. It was found that VSMCs also release vesicles rich in Ca^2+^ [64]. Anchorage-dependent cells, such as endothelial cells and VSMCs, depend on intercellular and cell–ECM contacts for survival. Integrin has been shown to promote cell survival by inhibiting cell death pathways and activating pro-survival factors such as nuclear factor kappa-light-chain-enhancer of activated B cells (NF-κB). The downregulation of vascular endothelial cadherins can induce apoptosis, while the interaction of VSMCs with tenascin C via integrins can prevent apoptosis by changing cell shape and inducing the clustering of epidermal growth factor receptors [66]. Cell–ECM interactions are crucial for apoptotic signaling pathways. Fibronectin signaling through focal adhesion kinase promotes cell survival. Several factors, including mechanical stimulation and growth factors, influence cell survival by activating pathways that prevent apoptosis. Different types of cell death, such as necrosis, apoptosis, and autophagy, affect disease progression in atherosclerosis. Endothelial cell apoptosis is common in the early stage, while VSMC and macrophage apoptosis are common in vulnerable lesions. Factors such as TRAIL (tumor necrosis factor-related apoptosis-inducing ligand), FGF21 (fibroblast growth factor 21) and antiapoptotic factors regulate apoptosis in vascular cells [67,68]. The regulation of VSMC turnover and apoptosis by miRNAs (e.g., miR-21, miR-26a, and miR-29b) and long noncoding RNAs (lncRNAs) (e.g., taurine upregulated gene 1) that target growth factor pathways and other factors may have therapeutic implications in the treatment of atherosclerosis [69,70]. Understanding the molecular mechanisms of cell survival and death in cardiovascular disease may help develop targeted therapies to prevent vascular death and related pathologies.

An association between calcification and apoptosis was also observed in articular cartilage. To prevent mineralization, it is necessary to halt the process of chondrocyte differentiation before hypertrophy. A study related to nitric oxide-induced apoptosis of articular chondrocytes revealed the development of ABs containing ALP with the ability to precipitate calcium phosphate crystals [23]. Throughout endochondral ossification, hypertrophic chondrocytes of the epiphyseal plate gain greater Ca^2+^ levels than proliferative chondrocytes [71]. Additionally, hypertrophied chondrocytes undergo apoptosis [72]. The concentration of Ca^2+^ in the vicinity of the membrane of chondrocytes at the final stage of differentiation was high, and the authors suggested that Ca^2+^ was incorporated into MVs or Abs [73]. Although it is hypothetical that the release of MVs capable of mineralization by chondrocytes is partly due to apoptosis, it has been demonstrated that the inhibition of apoptosis leads to a significant decrease in mineralization in cell cultures of VSMCs [41]. Studies of human and animal chondrocytes have highlighted the influence of various risk factors on cartilage degeneration, such as joint injuries, genetics, age, and obesity. Changes in matrix components (e.g., fibronectin, collagen type II, hyaluronan, and aggrecan) lead to cell death and perpetuate a cycle of cartilage degradation driven by enzymes such as MMP-13 and a disintegrin and metalloproteinase with thrombospondin motifs (ADAMTS) 4 and 5 [74]. The accumulation of advanced glycation end products also contributes to cartilage damage associated with high MMP-3 synthesis [75]. Changes in the cartilage ECM affect chondrocyte adhesion and survival, and integrins play a role in preventing cell death, while cleaved type II collagen induces chondrocyte apoptosis [76]. The inflammatory cytokines TNF-α and IL-1β produced by chondrocytes induce cell death and cause mitochondrial dysfunction. Mitochondria play an important role in cell function, and changes in their structure and function precede cell death under pathological conditions. In addition, miRNAs (e.g., miR-139, miR-10a-5p, and miR-146) and lncRNAs have been implicated in the regulation of chondrocyte apoptosis and cartilage diseases [66].

(C) The presence of circulating nucleation complexes. Nucleation complexes represent a calcification mechanism that is mostly modulated by the OPG–RANK–RANKL system (osteoprotegerin, a receptor activator of NF-κB, a receptor activator of NF-κB ligand). RANKL is an osteoblastic transmembrane protein that binds to its specific receptor RANK, a member of the TNF superfamily present in preosteoclasts and osteoclasts. The RANKL–RANK system stimulates osteoclastogenesis, whereas OPG (a member of the same TNF superfamily that is synthesized by osteoblasts) opposes this process. Because knockout mice with a deletion of the OPG gene have developed osteoporosis accompanied by severe media calcification of the aorta and renal artery, OPG is considered the main performer in the OPG–RANK–RANKL system [77]. OPG inhibited the arterial calcification induced by warfarin and vitamin D in rats [78]. Thus, we can envisage some of the links between the osteoclastic activity of bone remodeling and vascular calcification. It is acknowledged that RANKL enhances calcification of VSMCs by interacting with RANK and promoting BMP-4 production via activation of the alternative NF-κB pathway. Due to the evidence that RANKL promotes calcification of VSMCs, specific agents such as denosumab, a fully human monoclonal antibody, have been studied for their potential to prevent vascular calcification [79]. Most likely, the major determinant of bone metabolism is the OPG/RANKL ratio. Various factors influence the regulation of the RANKL–RANK–OPG system, such as certain cytokines (TNF-a, IL-1, IL-6 and IL-17), hormones (estrogen, vitamin D, and glucocorticoids), and growth factors [80]. Price et al. [81] suggested that the mineralization of soft tissue was induced by nucleation crystals generated in areas of bone resorption, which circulate through the bloodstream until they attach to tissues where they initiate calcification. In rats, Price et al. [82] discovered a complex consisting of phosphates, Ca^2+^, fetuin A and MGP (insoluble), which was released into the bloodstream from bone, thus explaining the blood transport of MGP. This complex was not found in humans [83].
(D) Imbalance/decline of inhibitors. An increasing number of ectopic calcification inhibitors are being identified using knockout mice or rat models. The presence of these inhibitors in body fluids and tissues saturated with calcium phosphate ions explains why soft tissues do not calcify instantly. Inhibitors of EM mineralization include OPG; osteopontin (a glycoprotein secreted by osteoblasts and involved in bone remodeling); Ank (a transporting transmembrane protein that controls extracellular PPi export); phosphodiesterase nucleotide pyrophosphatase (an extracellular PPi-generating ectoenzyme) [43]; MGP (involved in the clearance of calcium phosphate but also an inhibitor of osteogenic differentiation of VSMCs in different pathologies) [84,85,86]; fetuin A (a circulating inhibitor of calcification that inhibits de novo formation of HC, but has no effect on the already formed crystals) [87]; and Smad 6 (Smad 6 null mice developed extensive vascular wall calcification and cartilaginous metaplasia in the aorta due to suppression of the BMP signaling pathway) [88].

In addition, parathyroid hormone and vitamin D exert adverse effects on calcification. Vitamin D3 stimulates the intestinal absorption of Ca^2+^ and mobilization of Ca^2+^ from bones, but also stimulates the influx of Ca^2+^ in VSMCs and inhibits their proliferation (VSMCs express receptors for vitamin D). A study on VSMC cell cultures conducted by Jono et al. [89] showed that vitamin D3 promoted the deposition of Ca^2+^ by inhibiting the expression of parathyroid hormone-related peptides. The study also demonstrated that parathyroid hormone and parathyroid hormone-related peptide inhibited the calcification of VSMCs by decreasing ALP activity.

Torres [90] and Speer et al. [42] broadly circumscribed the modulating factors of vascular calcification as follows (see Table 2).

As a factor that promotes calcification, Pi regulates MGP expression in osteoblasts by stimulating both messenger ribonucleic acid (mRNA) and protein synthesis [91]. Moreover, heat shock protein 70 (a stress-inducible protein expressed in atherosclerotic lesions) binds to MGP and stimulates the activity of BMP-4; thus, heat shock protein 70 is considered a potential link between cellular stress, inflammation, and signaling via BMP [92]. We could also mention klotho, a β-glucuronidase whose genetic variants have been associated with aging [93].

Since physiological levels of calcium and phosphate are close to their solubilization constant in serum, it has been hypothesized that under basic conditions, inhibitors are required to prevent ectopic calcification. Thus, diminished local/serum levels of inhibitors or an imbalance in their activity leads to ectopic calcification.

## 4. Conclusions

Cells play an important role in the mineralization process, which involves the deposition of inorganic substances that are essential for bone formation. However, pathological mineralization can negatively affect the cardiovascular system and joints, causing disease and complications, and factors such as hormonal imbalance and oxidative stress also contribute to the development of ectopic calcification.

Abnormal calcification of soft tissues is a complex process influenced by several factors. Ectopic calcification resembles normal bone mineralization, and factors such as cell type, cell interactions, EM composition, and mechanical forces can influence the effects of calcification. The incidence of this phenomenon is becoming ubiquitous due to increased life expectancy and chronic degenerative diseases, which affect elderly people and cause joint and cardiovascular complications.

Additional information on MV function may allow the identification of effective targets for the development of new therapeutic drugs for the treatment of bone and vascular mineralization. There has been significant progress in our knowledge of how MVs are formed and secreted within cells. While understanding the specific process of MV formation is challenging, it could be feasible to enhance bone formation by encouraging the body’s own production of MVs. In addition, after the MVs are successfully purified, they can be utilized as biological substances to stimulate the regeneration of hard tissue. Methods to decrease programmed cell death and support atherosclerotic plaques in arteries could involve blocking caspases and regulating MMPs. Because caspases are crucial for apoptosis, their inhibition could contribute to minimizing apoptotic cell death and promoting the stability of atherosclerotic plaques. Another approach for managing ectopic calcification could involve manipulating the OPG–RANK–RANKL system to develop new therapeutic strategies.

We encompassed here the similarities and differences between vascular and cartilage calcifications and their relationship to physiological osteogenesis and pathological calcifications, identifying a few therapeutic approaches. Understanding the complex processes and factors involved in ectopic calcification can help develop strategies to prevent and treat diseases and complications associated with this condition.

## Figures and Tables

**Table 1:** Comparison between bone formation and vascular calcification.

Bone Formation	Micro/Macrostructural Characteristics	Mechanism of Mineralization	Localization
Intramembranous ossification	- Without cartilage matrix - Rich vascularization - Collagen type I in EM	- MV-dependent calcification - Direct EM calcification rich in type I collagen fibers	- Formation of bones: frontal, parietal, occipital, partially temporal, clavicle, mandible, maxilla - Odontogenesis (dentin)
Endochondral ossification	- Initiation on an avascular cartilaginous template - Collagen type II EM → type X → type I - Cartilage calcification → neovascularization, replacement of calcified cartilage with bone tissue	- MV-dependent calcification - Hypertrophic chondrocyte apoptosis	- Formation of bones: base of the skull, vertebrae, long bones and extremities of the bones - Healing process of fractures
**Vascular Calcification**	**Micro/Macrostructural Characteristics**	**Mechanism of Mineralization**	**Associated Pathology**
Intima calcifications	- Evolution of lipid streaks (fatty streak) and fibrous plaque - Macrophages, T lymphocytes, endothelial dysfunction, activation of platelets and myofibroblasts - Media → intima migration of VSMCs - Eccentric, intraluminal protrusion	- Calcium is deposited along with lipoproteins and phospholipid cellular debris - Chondrogenic metaplasia (endochondral ossification) - Cell apoptosis, necrosis and cell debris	- Atherosclerosis - Hypercholesterolemia
Media calcifications (Mönckeberg’s sclerosis)	- Inflammation/activation of adventitia - Macrophages, T lymphocytes, myofibroblasts, adipocytes, VSMCs and calcified medial vascular cells - Circumferential, decreased vascular compliance	- MV-dependent calcification - Osteogenesis similar to intramembranous ossification - Insignificant/no cartilage formation	- DM type I and II - End-stage renal disease - Elderly people
Calcifications of heart valves	- Macrophages, T lymphocytes, interstitial adipocytes and myofibroblasts - Inflammation/activation of interstitial cells	- Dystrophic calcium deposition - High frequency of osteogenic calcifications, lamellar bone formation - Low frequency of chondrogenic (endochondral ossification)	- Calcified senile aortic sclerosis - Bicuspid aortic valve calcifications - Bioprosthetic valve calcifications
Vascular calciphylaxis (Calcific uremic arteriolopathy)	- Systemic calcification of organs and soft tissues; subcutaneous calcification - Occurs when the physiological solubilization threshold of calcium phosphate is exceeded (>60 mg^2^/dL^2^)	- Amorphous calcium phosphate deposit - Increased serum Ca x PO4 - Without osteogenesis and chondrogenesis	- Chronic/acute renal failure - Secondary hyperparathyroidism - Hypercoagulable state - Tumor lysis syndrome - Iatrogenic hyperphosphatemia

Abbreviations: VSMCs, vascular smooth muscle cells; DM, diabetes mellitus; EM, extracellular matrix; MVs, matrix vesicles.

**Table 2:** Factors modulating ectopic calcification.

Protective Factors of Calcification	Favoring Factors of Calcification
osteopontin	TNF-α
OPG	TGF-β
fetuin-A	oxidized and acetylated LDL-C
osteonectin	C-reactive protein
MGP	leptin
BMP-7	BMP-2
Mg^2+^	advanced glycation end-products
PPi	Pi
↓ Ca x PO_4_	↑ Ca x PO_4_
vitamin K	interleukin-4
HDL-C	interleukin-6
growth arrest-specific protein 6	glucocorticoids
albumin	ROS
parathyroid hormone	collagen tip I
parathyroid hormone related peptide	fibronectin
phosphonoformic acid	25-OH colesterol
natriuretic peptide type C	17β-estradiol
adrenomedulin	Ca^2+^
	uremic serum
	1, 25-dihydroxycholecalciferol
	cyclic adenosine monophosphate

Abbreviations: OPG, osteoprotegerin; MGP, matrix Gla protein; BMP-7, bone morphogenic protein 7; PPi, inorganic pyrophosphate; HDL, high-density-lipoprotein cholesterol; TGF-β, transforming growth factor β; TNF-α, tumor necrosis factor α; LDL-C, low-density-lipoprotein cholesterol; BMP-2, bone morphogenic protein 2; Pi, inorganic phosphate; ROS, reactive oxygen species; 25-OH cholesterol, 25-hydroxycholesterol; ↓, decreases; ↑, increases.
